# Algorithm for Reporting Free Hemoglobin in ECMO Patients: Need for a Multidisciplinary Approach

**DOI:** 10.3390/jcm15020867

**Published:** 2026-01-21

**Authors:** Ivana Baršić Lapić, Ljiljana Zaninović, Daniel Lovrić, Ana Lončar Vrančić, Dora Rebrek, Dunja Rogić

**Affiliations:** 1 Department of Laboratory Diagnostics, University Hospital Center Zagreb, 10000 Zagreb, Croatia; ljiljana.zaninovic@kbc-zagreb.hr (L.Z.); ana.loncar.vrancic@kbc-zagreb.hr (A.L.V.); dora.rebrek@kbc-zagreb.hr (D.R.); drogic@kbc-zagreb.hr (D.R.); 2 Intensive and Acute Cardiac Care Unit, Department for Cardiovascular Diseases, University Hospital Center Zagreb, 10000 Zagreb, Croatia; daniel@dlovric.net

**Keywords:** plasma free hemoglobin, ECMO, hemolysis index, icteria, algorithm

## Abstract

**Background:** Intravascular hemolysis is a common complication in patients undergoing extracorporeal membrane oxygenation (ECMO), with plasma free hemoglobin (pfHb) serving as a biomarker for detection. Without standardized protocols, laboratories face challenges in interpreting and reporting results. Hemolysis indices may enhance reporting accuracy. **Methods:** This retrospective observational study at University Hospital Center Zagreb included 61 lithium heparin plasma samples from ECMO patients. pfHb was measured using the Harboe method (fHb) and estimated from hemolysis indices on Abbott Alinity c analyzer (efHb). Total and conjugated bilirubin, hemolysis, icterus, and lipemia indices (HIL) were recorded. Method comparison used Passing-Bablok regression and Bland–Altman analysis. An algorithm for pfHb reporting accounting for HIL interferences was developed. **Results:** Significant differences were observed between methods, with Harboe yielding higher median fHb (261 mg/L) versus efHb (58 mg/L). Regression analysis showed constant negative bias of −91 mg/L (95% CI: −143 to −16) for efHb relative to fHb. Bland–Altman analysis demonstrated wide limits of agreement. Correlation between fHb and efHb was moderate (Spearman’s rho = 0.618, *p* < 0.001). The delta between methods increased with higher bilirubin concentrations. An algorithm integrating HIL indices with the Harboe method was developed to guide result validation and reporting. **Conclusions:** Accurate hemolysis assessment in ECMO patients requires careful interpretation, appropriate method selection, and laboratory–clinician collaboration. The proposed algorithm improves the clinical utility of pfHb testing by accounting for analytical interferences and supporting informed decision-making.

## 1. Introduction

Extracorporeal membrane oxygenation (ECMO) is increasingly used in modern intensive care as life-saving support for severe cardiac and respiratory failure. It can support or replace lung function by providing gas exchange in a veno-venous circuit (V-V ECMO) or provide circulatory support with gas exchange in the case of a failing heart with or without failing lungs when used as a veno-arterial circuit (V-A ECMO) [1]. Although ECMO has been in existence since the 1970s, early application was plagued by high complication rates. In the last three decades, improved risk-benefit profiles have enabled longer-term support for organ recovery or as a bridge to transplantation [2].

Hemolysis during ECMO primarily results from non-physiological shear stress that is generated as blood is propelled through the rotational pump, cannulas, tubing, and the oxygenator. Excessive rotational speeds, high flow rates relative to cannula size, turbulence, and abrupt pressure gradients can disrupt red blood cell membranes, leading to intravascular hemolysis. Additional contributing factors include cannula malposition, clot formation within the circuit, pump head thrombosis, and oxygenator dysfunction, all of which further increase mechanical stress on erythrocytes. Clinically, ECMO-related hemolysis is relevant because it is associated with inflammation, acute kidney injury, coagulation disturbances, and worse outcomes, underscoring the importance of meticulous circuit management and early recognition [3,4]. It is also more often present in non-survivors compared to survivors [5,6], and is more prevalent in V-A ECMO than in V-V ECMO due to smaller diameter cannulas usually being employed as well as a need for higher flow rates to support systemic circulation [7].

Extracorporeal Life Support Organization (ELSO) recommends monitoring plasma free hemoglobin levels (pfHb). When pfHb exceeds 50 mg/dL, investigation for the underlying cause is recommended. When high pfHb levels are detected, ELSO suggests avoiding excessive negative inlet (suction) pressure and high resistance in the return limb, while specifically evaluating for circuit clotting and oxygenator thrombosis [8]. Hemolysis is usually monitored by measuring pfHb. However, standardized monitoring protocols for ECMO are lacking. Both the definitions of hemolysis and diagnostic cut-off values vary across studies, limiting the ability to determine its true clinical incidence [6]. A recent survey of ECMO centers found that while 68% report having a standard protocol for hemolysis monitoring, the specific protocols vary widely [9].

Several studies suggest that measuring the hemolysis indices on a biochemistry analyzer can replace the Harboe method, which is considered the reference method for measuring pfHb [10,11]. The Harboe method requires manual sample handling and measurements at three specific wavelengths (380 nm, 415 nm, 450 nm) by skilled operators using an independent spectrophotometer. The method is prone to interference from high bilirubin concentrations and lipid particles, which can be present in samples of ECMO patients. Hemolysis, icteria, and lipemia indices (HIL indices) are measured on a biochemistry analyzer. Their calculations are based on absorbance measurements that provide (semi)quantitative estimates of hemolysis, icterus, and lipemia/turbidity [12]. Unlike bilirubin or hemoglobin, no substance can be used to mimic the physical and chemical interfering properties of lipemic samples. Lipemia indices on biochemistry analyzers are established with Intralipid^®^ or the equivalent materials. However, lipemia has a heterogeneous nature so Intralipid^®^ completely misses the range of values for large VLDL and misses the lower and upper ranges for chylomicrons [12]. This is the reason for the difficulty in simulating lipemic samples. For icteria indices many studies are conducted using unconjugated bilirubin and ditaurobilirubin conjugate. While this compound often accurately reflects the effects of conjugated bilirubin it may not always produce the same effects as naturally occurring forms of bilirubin.

The aim of this study is to show the correlation between pfHb measured by the Harboe method and plasma free hemoglobin estimated by indices of hemolysis. Additionally, it shows how different concentrations of total and conjugated bilirubin affect this correlation. This study also provides an algorithm for reporting pfHb values for ECMO patients that can also be used for all other patients requiring pfHb measurements.

## 2. Materials and Methods

### 2.1. Study Design

We conducted a retrospective observational study at the Department of Laboratory Diagnostics, University Hospital Center Zagreb, Croatia. This study utilized data from 61 samples initially collected in the intensive care unit from patients on ECMO. Samples were then transported to the clinical laboratory for routine biochemistry analysis. Samples were processed according to standard laboratory protocols, and laboratory measurements were performed as part of routine diagnostic workflows. No additional sample collection or intervention was needed.

All samples were collected in lithium heparin tubes (Greiner Bio-One, Kremsmünster, Austria) and centrifuged for 10 min at 4000 rpm. Plasma was then separated into two aliquots. One aliquot was used to measure total and conjugated bilirubin concentrations, as well as HIL indices. The second aliquot was centrifuged again before determining fHb concentration. The data of free hemoglobin, total and conjugated bilirubin concentrations (tBil and dBil), as well as indices of hemolysis, lipemia, and icteria were collected.

Analyses of fHb were performed on a BioMate 3 spectrophotometer (Thermo Fisher Scientific, Waltham, MA, USA) using the Harboe method [13]. It involves measuring absorbance at three distinct wavelengths, 380 nm, 415 nm, and 450 nm. The fHb concentration is then calculated using the equation:

fHb (mg/L) = 836 × (2 × A415 − A380 − A450).

A415, A380 and A450 represent the absorbances at 380 nm, 415 nm, and 450 nm, respectively. This method utilizes the specific absorbance spectra of free hemoglobin, oxyhemoglobin, and methemoglobin to accurately quantify plasma free hemoglobin.

Analyses of the HIL indices and the concentration of total and conjugate bilirubin were performed on an Abbott Alinity c, fully automated analytical system (Abbott Laboratories, Chicago, IL, USA). The Abbott Alinity c analyzer measures HIL indices using a specific absorbance at four wavelength pairs for each index (500 nm/524 nm; 572 nm/604 nm; 628 nm/660 nm; 524 nm/804 nm). To determine the concentrations of total and conjugated bilirubin, the Alinity c Direct Bilirubin Reagent Kit (Ref.number: 07P97) and Total Bilirubin2 Reagent Kit (Ref.number: 04U05) were used. The HIL indices were measured on the Alinity c analyzer, with a 0.9% sodium chloride (NaCl) solution. The relative concentration of interferents is calculated mathematically. One unit of the hemolysis index is approximately equivalent to 10 mg/L of free hemoglobin concentration.

### 2.2. Statistical Analysis

Statistical analysis was performed using MedCalc^®^ Statistical Software version 23.4.0 (MedCalc Software Ltd., Ostend, Belgium). For each analyte in the study, the median and the *p* value were calculated. Normality of data distributions was evaluated using the Shapiro–Wilk test. A *p*-value of <0.05 was considered statistically significant for all analyses. Correlation analysis was conducted as follows:

1. Between fHb and efHb
2. Between fHb and both total and conjugated bilirubin concentrations.
3. Between efHb and both total and conjugated bilirubin concentrations.

Additionally, regression analysis was employed for further investigation of relationships between analytes and to assess the impact of bilirubin values on fHb. Passing–Bablok linear regression and Bland–Altman analysis were used to assess differences between the compared results. A multi-line graph was utilized to illustrate the delta value between fHb and efHb in relation to the concentrations of tBil and dBil.

## 3. Results

### 3.1. Characteristics of the Sample

The median range of values and *p* value for each measured analyte are presented in Table 1. The comparison between fHb and efHb shows significant differences in their distribution and values. The median of fHb is much higher (261 mg/L) than the median of efHb (58 mg/L) and the 95% confidence intervals (95% CI) do not overlap. This indicates a substantial difference in central tendency between the two measurement methods.

### 3.2. Correlations Between fHb and efHb

**Figure 1:**
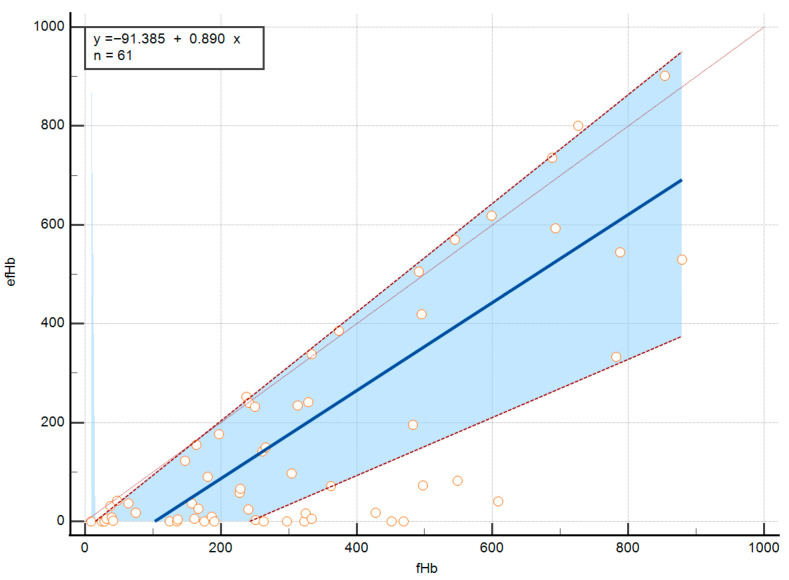
Passing-Bablok regression analysis.

Detailed data on method comparison comprising Passing–Bablok regression are presented in Table 2 and are graphically shown in Figure 1. The Passing-Bablok regression, with efHb plotted as the dependent variable against fHb as the reference method, revealed a constant systematic difference between the two methods. Since the 95% CI for the intercept does not include zero, this indicates that efHb systematically underestimates pfHb by approximately 91 mg/L compared to the Harboe method at the lower end of the measurement range. The 95% CI for slope includes 1, so there is no statistically significant proportional difference between the two methods. Spearman’s rank correlation coefficient (rho) of 0.618 with *p* < 0.001 indicates a moderate positive correlation between fHb and efHb values.

**Figure 2:**
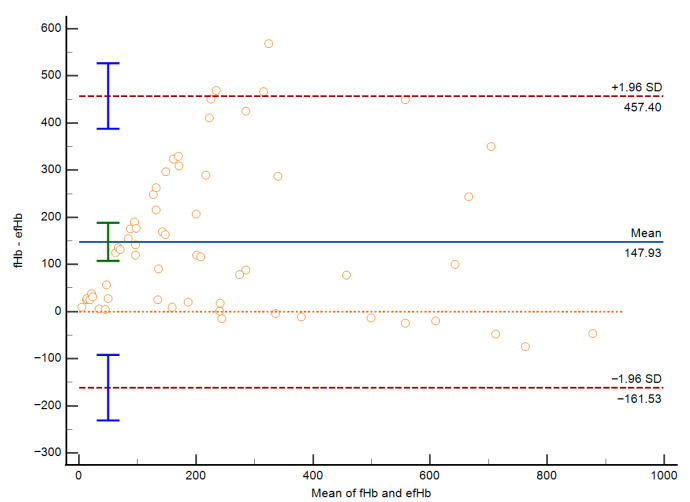
Bland–Altman analysis.

Bland–Altman analysis (Figure 2) shows the mean bias between the two methods of 147.93 mg/L, with a 95% CI from 107.50 to 188.37 mg/L indicating that on average, the Harboe method measures pfHb values about 148 mg/L higher than the hemolysis index estimation. The 95% limits of agreement range from −161.53 mg/L to 457.40 mg/L, demonstrating substantial variability between the two methods for individual measurements. Importantly, the magnitude of disagreement between methods was strongly influenced by total bilirubin concentration.

### 3.3. Correlations Between fHb and efHb Depending on Total and Conjugated Bilirubin Concentration

**Figure 3:**
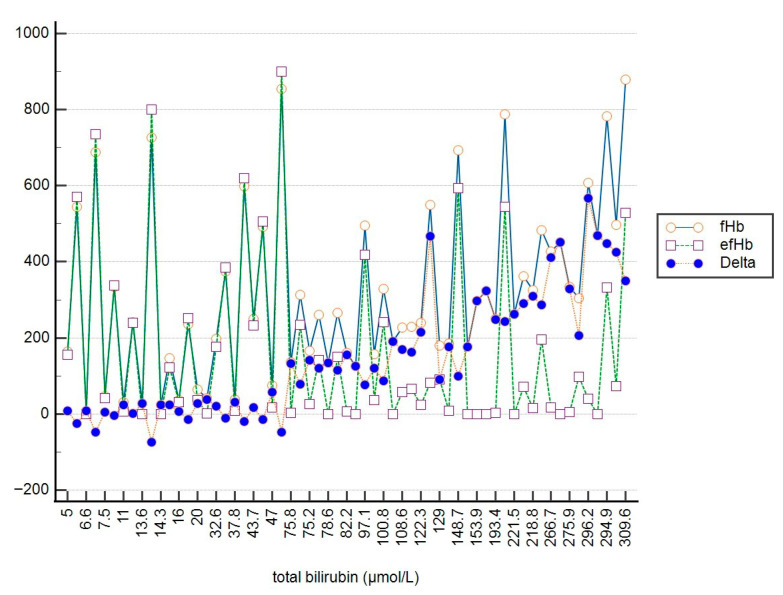
The change in delta values between fHb and efHb varies depending on the concentration of total bilirubin.

**Figure 4:**
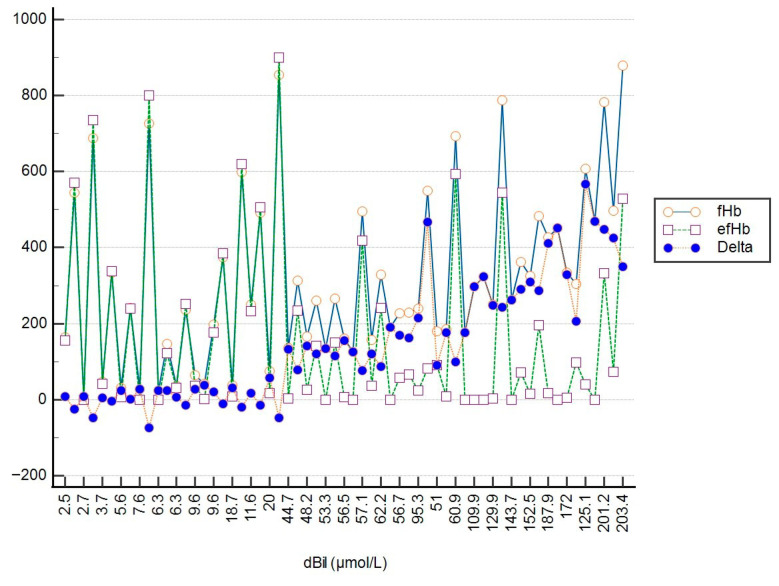
The change in delta values between fHb and efHb varies depending on the concentration of conjugated bilirubin.

To illustrate the relationship between fHb, efHb, and tBil, as well as between fHb, efHb, and dBil, a multi-line graph was utilized (see Figure 3 and Figure 4). The x-axis represents bilirubin concentration (µmol/L), while the y-axis shows fHb and efHb concentrations (mg/L). The graph displays three distinct data series: fHb (orange circles), efHb (purple squares), and their differences (Delta, blue circles).

At lower tBil and dBil concentrations (tBil < 75 μmol/L; dBil < 44 μmol/L), the values of fHb and efHb are very similar, meaning that both methods for determination of pfHb provide equal clinical information about the level of hemolysis within this range of bilirubin concentrations. As bilirubin concentrations increase, the trend shows a difference between fHb and efHb. The values of efHb are much lower than the values of fHb, likely reflecting interference with the Harboe method.

### 3.4. Algorithm for Analysis and Reporting Results of fHb

**Figure 5:**
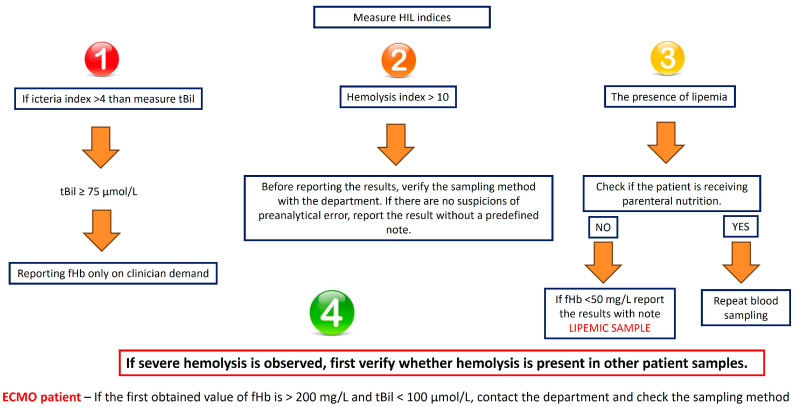
Algorithm for reporting results of fHb: Step 1—Evaluate icterus interference: if icteric index > 4, measure total bilirubin; if tBil ≥ 75 μmol/L, use Harboe method (fHb) only upon clinician request due to significant bilirubin interference. Step 2—Assess hemolysis index: if >10, verify sampling method with the clinical department to exclude preanalytical hemolysis before reporting. Step 3—Check for lipemia interference: if present and patient receives parenteral nutrition, request new sample after temporary discontinuation; if fHb < 50 mg/L and lipemia is present without parenteral nutrition, report with “LIPEMIC SAMPLE” notation. Step 4—In severe hemolysis cases, always verify findings in other patient samples to distinguish true intravascular hemolysis from preanalytical error.

According to the previously mentioned results, we designed the algorithm for reporting results of pfHb combining HIL indices and the Harboe method (see Figure 5). This algorithm includes reporting only on fHb and not on efHb. However, efHb can aid in decision-making when final results are reported.

After centrifugation, the HIL indices are measured. If the icterus index exceeds 4, the concentration of tBil is determined (one unit of the icterus index corresponds to approximately 69 μmol/L of total bilirubin). If tBil is equal to or greater than 75 μmol/L, the reporting of fHb results should be limited to cases where there has been prior communication with the clinician and upon their request. The tBil cut-off value of 75 μmol/L was derived empirically from our dataset by analyzing the delta (absolute difference) between fHb and efHb across varying total bilirubin concentrations. We observed that at total bilirubin concentrations above 75 μmol/L, the relative difference between methods exceeded more than 50%, indicating clinically significant analytical interference. Below this threshold, the interference remained within acceptable analytical limits. This cut-off therefore represents the inflection point where bilirubin interference substantially compromises the accuracy of the Harboe method.

It is important to communicate with the clinician about the potential interference caused by elevated bilirubin levels, so they can appropriately integrate this information into the patient’s clinical assessment. In such a case repeating the sampling is not recommended because the icterus index will not change with the new sampling.

The next step is to check the hemolysis index. If it exceeds 10 (approximately 100 mg/L fHb), it is necessary to check how venipuncture was performed to exclude possible preanalytical errors before reporting any results. In cases where lipemia is present, the patient’s parenteral nutrition status should be assessed, as well as sedation with propofol that can cause lipemia when high doses are employed. If the patient is receiving parenteral nutrition and it is possible to temporarily discontinue the nutrition—and in case of propofol, if it is possible to adjust the sedation protocol—a new sample should be requested. If discontinuation is not possible and the fHb concentration is below 50 mg/L, results may be reported with an annotation indicating the sample was lipemic.

Furthermore, if the initial value of fHb exceeds 200 mg/L while the tBil concentration is below 100 μmol/L, the sampling method should be reviewed to ensure that poor phlebotomy technique is not contributing to an artificially elevated value.

This algorithm can ensure better understanding of the obtained value of fHb in the presence of common interferences, thereby optimizing clinical decision-making.

## 4. Discussion

Although there are no standardized hemolysis monitoring protocols for patients on ECMO, pfHb is still the most used biomarker for the degree of hemolysis. Elevated levels of pfHb will lead to clinical decisions, usually prompting lower flows and higher anticoagulation targets. These adjustments are associated with less hemodynamic support and a higher risk of bleeding. Therefore, having a precise estimation of hemolysis is crucial in a successful ECMO program. Patients who present with elevated bilirubin on ECMO therapy have an especially unfavorable prognosis [14]. In these cases, knowing exactly how much hemolysis is occurring becomes critical.

Both methods for measuring pfHb—the Harboe method and the hemolysis indices—are used today. However, both methods have some limitations and cannot be used interchangeably. It is important to note that different manufacturers do not use the same substances to determine indices of hemolysis, icterus, and lipemia. They differ based on the interferent material used and the maximum concentration tested [12]. To establish icterus indices, manufacturers use various approaches. Some use only unconjugated bilirubin, while others employ a combination of unconjugated and conjugated bilirubin. Still others use combinations of bilirubin forms with patient samples. For hemolysis indices, erythrocyte hemolysate or a combination of erythrocyte hemolysate and patient samples are used. Due to a lack of standardized lipoprotein reference materials Intralipid^®^ is commonly used, even though it does not have the characteristics of native lipemic samples. Cross-interferences among hemolysis, icterus, and lipemia indices are possible. Although both manufacturers and laboratories test for these interferences, the substances used to develop the HIL indices may not accurately represent real patient samples. As a result, when estimating pfHb, it is challenging to determine the exact concentration of hemoglobin accurately.

Because of the nature of these indices, HIL index results should be considered qualitative or as estimates of the degree of hemolysis, icterus, and lipemia. Our study highlights complementary roles: the Harboe method provides precise quantitative free hemoglobin measurement, while HIL indices offer quick, integrated sample hemolysis screening in ECMO patient monitoring.

Several studies have investigated hemolysis index as an alternative to direct measurement of pfHb concentration but they are different designs from ours. Zapletal et al. compared free plasma hemoglobin and hemolysis index obtained on the Roche Cobas system [11]. They concluded that hemolysis index has an excellent correlation with plasma free hemoglobin in both patients with and without ECMO. However, it has a low positive predictive value to be used as a diagnostic test. Bürki et al. compared different analytical systems—Roche Cobas and Abbott Architect—with the manual spectrophotometry method, different from the Harboe method [15]. They concluded that lipemia causes Roche Cobas to overestimate and causes Abbott Architect to underestimate free hemoglobin.

Our results show that bilirubin levels significantly impact the reliability of pfHb results obtained with the Harboe method. Additionally, we assert that completely transitioning from the Harboe method, as a reference method, to measuring the hemolysis index may not always be the safest approach for assessing the degree of hemolysis in ECMO patients. In spectrophotometric methods we should always be aware of possible interferences that can alter the true value of the measured analyte. The novelty of our study lies in our demonstration, using real patient samples, that bilirubin significantly interferes with the measurement of pfHb. We have determined the cut-off values for total and conjugated bilirubin at which the Harboe method and hemolysis index start to show notable differences. Recognizing lipemia as a source of interference in the Harboe method and in hemolysis index determination on automated analyzers, as well as the limitations of using Intralipid^®^ to simulate lipemia interference, we developed an algorithm to improve the reliability of the reported analysis results.

Algorithms can be implemented in clinical practice leading to modification in nutrition and sedation regiments to obtain a non-lipemic sample. If there is a high suspicion of hemolysis, clinicians can adjust the level of ECMO support as well as anticoagulation resulting in significantly lower flow and higher anticoagulation targets. Since that decision can influence outcomes significantly, it should not be taken lightly. In a clinical setting the ECMO system is regularly checked for thrombosis and oxygenator disfunction. High plasma free hemoglobin present along with findings of small thrombi in the oxygenator and less than ideal gas exchange would lead to earlier exchange of the system.

To our knowledge, this is the first study to propose an algorithm for reporting pfHb results that includes cut-off values for total and direct bilirubin, along with lipemia assessment. We also address the risk of falsely elevated values caused by preanalytical sampling errors. Limitations of this study include its retrospective design and the single-center setting, which may limit applicability. Additionally, while the sample size allowed demonstration of significant correlations, larger prospective studies are warranted to validate and refine the proposed algorithm and to explore its impact on clinical outcomes. In our study we did not collect data on individual patient identifiers. Therefore, we cannot exclude the possibility that multiple samples from the same patient were included. If present, such repeated measurements could introduce clustering effects that violate statistical independence assumptions. However, this was a method comparison study focused on analytical performance under various interference conditions rather than patient-level clinical outcomes. Each sample represents an independent analytical measurement under the conditions present at the time of analysis, including varying degrees of hemolysis, bilirubin, and lipemia interference. This study did not employ multivariable regression modeling with confounder adjustment, as our analytical approach focused on method comparison rather than predictive or causal modeling. Future studies examining the clinical impact of hemolysis measurement methods may benefit from multivariable approaches that account factors such as ECMO duration, circuit characteristics, and clinical outcomes.

The proposed algorithm was developed using the Abbott Alinity c analyzer. Hemolysis index values and interference thresholds vary substantially between analyzer platforms due to differences in wavelength selection, calibration, and calculation algorithms. Therefore, the specific cut-off values reported here should be considered analyzer-specific. They require validation before implementation on alternative platforms. Each laboratory should establish its own thresholds using similar methodology with paired spectrophotometric measurements.

Although ECMO is now considered a routine procedure, its management requires a multidisciplinary approach. pfHb is just one component in the monitoring of both the ECMO process and the patient’s status. Our comparative analysis of the Harboe method, the hemolysis index, and the concentrations of total and conjugated bilirubin, shows that reporting free hemoglobin results requires close collaboration between the laboratory and clinicians. This collaboration is crucial for accurately addressing any concerns about the reliability of the results. It enables clinical validation based on the patient’s condition and the ECMO procedure. It also supports timely adjustments in patient management and ECMO protocols.

## Figures and Tables

**Table 1:** Characteristics of the sample, median, and range of values for each analyte.

Analyte	Sample Size	Median (95% CI *)	*p* Value **	Range of Values
fHb, mg/L	61	261 (209–327)	0.001	9–879
efHb, mg/L	61	58 (20–134)	<0.001	0–901
tBil, μmol/L	61	82 (46–118)	<0.001	5–313
dBil, μmol/L	61	53 (19–65)	<0.001	2–212

* 95% confidence interval; ** Shapiro–Wilk test.

**Table 2:** Results of method comparison between fHb and efHb. Intercepts and slopes with 95% CI according to Passing-Bablock regression analysis, and Spearman’s coefficient of rank correlation, are presented.

	Intercept	95% CI for Intercept	Slope	95% CI for Slope	Spearman’s Coefficient of Rank Correlation (Rho)	*p* Value	95% CI
fHb/efHb	−91.39	−142.60-(−16.4706)	0.89	0.58–1.10	0.618	<0.001	0.43–0.75

## Data Availability

The data presented in this study are available on request from the corresponding author.
